# Dosimetric Evaluation of Incidental Irradiation to the Internal Mammary Chain After Surgery in Breast Cancer Patients

**DOI:** 10.3389/fonc.2022.839831

**Published:** 2022-03-02

**Authors:** Wei Wang, Tao Sun, Yingtao Meng, Min Xu, Yingjie Zhang, Qian Shao, Yuanfang Song, Jianbin Li

**Affiliations:** ^1^ Department of Radiation Oncology, Shandong Cancer Hospital and Institute, Shandong First Medical University and Shandong Academy of Medical Sciences, Jinan, China; ^2^ Department of Medical Physics, Shandong Cancer Hospital and Institute, Shandong First Medical University and Shandong Academy of Medical Sciences, Jinan, China; ^3^ Department of Radiation Oncology, Wei Hai Municipal Hospital, Cheeloo College of Medicine, Shandong University, Weihai, China

**Keywords:** breast cancer, radiotherapy, radical mastectomy, breast-conserving surgery, internal mammary chain incidental irradiation dose

## Abstract

**Background and Purpose:**

The low rate of internal mammary node (IMN) recurrence was attributed to systemic therapy and internal mammary chain (IMC) coverage by the tangential fields of irradiation. This study aimed to evaluate the incidental irradiation dose to the IMC in breast cancer patients after surgery and to estimate the clinical predictive parameters affecting the magnitude of the IMC.

**Materials and Methods:**

A total of 138 patients treated with postmastectomy radiotherapy and 210 patients undergoing radiotherapy after breast-conserving surgery (BCS) in our hospital were retrospectively analyzed. The mean dose (Dmean) to the IMC and the first to third intercostal spaces of IMC levels (ICS1–3) were evaluated. We evaluated the IMC coverage according to the type of surgery and whether the ipsilateral supraclavicular fossa (SCF) was included in the irradiation field.

**Results:**

The incidental radiation dose to the IMC was 29.69 Gy, and the dose delivered to the IMC, ICS1, and ICS2 showed a greater coverage in the modified radical mastectomy (MRM) group when compared with the BCS group (32.85 vs. 27.1 Gy, 26.6 vs. 12.5 Gy, 34.63 vs. 30.42 Gy). The dose delivered to ICS3 showed no difference between the MRM and BCS groups (37.41 vs. 36.24 Gy). Furthermore, 131 patients (37.64%) received radiotherapy to the chest wall and ipsilateral SCF. In the univariate analysis, both surgery type and SCF irradiation were parameters affecting the Dmean of incidental radiation to the IMC (*r* = −0.179, *P* = 0.001; *r* = −0.175, *P* = 0.001). In the multivariate analysis, surgery type was the only correlative factor that affected incidental radiation dose to the IMC (*r* = –3.534, *P* = 0.000).

**Conclusion:**

The real influencing factor of incidental dose to the IMC was the surgery form rather than the accession of SCF irradiation.

## Introduction

Adjuvant breast cancer radiotherapy reduces the risk of local/regional recurrence and improves overall survival (OS) of patients undergoing breast-conserving surgery (BCS) and mastectomy (1, 2). In 2016, the National Comprehensive Cancer Network published updated clinical practice guidelines, and the Royal College of Radiologists published a consensus statement that included internal mammary node (IMN) irradiation (IMNI) guidelines and practice changes (3, 4). IMNI was administered to patients with positive axillary lymph nodes (ALNs) and patients with medial or central breast cancers while ALN was negative.

When patients underwent three-dimensional (3D) treatment planning, the incidence and severity of radiation-induced lung injury and ischemic cardiac events were minimal and acceptable (5, 6). However, an ancillary result from the Korean Radiation Oncology Group 08-06 study has revealed that radiation pneumonitis (RP) increased in breast cancer patients (6.5% of patients who underwent IMNI vs. 3.3% of patients who did not undergo IMNI) with internal mammary chain (IMC) irradiation, and grade 2 RP was observed only in the IMNI group (5). In the Danish Breast Cancer Cooperative Group-IMN study, all patients with left-sided breast cancer were treated without IMNI (median follow-up period: 9.6 years), but a systematic review and meta-analysis proved that when IMNI was performed, patients with left-sided breast cancer were at a higher risk of cardiovascular (CV) death than those with right-sided breast cancer (6). This difference in CV mortality was more apparent after 15 years of follow-up (7). A 15-year analysis of the European Organization for Research and Treatment of Cancer (EORTC) 22922/10925 trial showed that IMC and supraclavicular fossa (SCF) lymph node chain irradiation significantly reduced breast cancer mortality and recurrence in patients with stage I–III breast cancer. However, this does not translate to improved OS, nor does it provide any indication of their late irradiation reactions (8).

The IMN metastasis rate for patients with positive ALN metastases was 28%–52%, whereas the metastasis rate for patients with IMN involvement of tumor with medial or central location was 32%–65% (9). Despite the high incidence of IMN involvement after primary breast cancer treatment, the overall recurrence rate in IMNs is <1.5% even when IMCs are not excised or irradiated (10–12). For patients with negative ALNs and one to three positive ALNs, the recurrence rate in IMN was <0.3% (9), and in the IMC irradiation group, the recurrence rate was 0.2% (10). The clinical outcomes of incidental radiation to regional lymph nodes in terms of locoregional control are gaining widespread attention (13–23). The exceptionally uncommon overall recurrence in the IMNs is associated with systemic treatment and incidental IMNI (10, 20).

We have proven that incidental irradiation dose to the IMC was not associated with radiotherapy technique, both for patients who underwent BCS and modified radical mastectomy (MRM) (21–23). Given the fact that the correlation identified between IMNI dose and surgical treatment is still unclear, our study evaluated the incidental irradiation of IMC drainage routes [first to third intercostal spaces (ICS1–3)] with no formal indication for irradiation of the IMC in a real-life cohort. First, the impact of the type of surgery on IMC coverage in breast cancer patients in China who did not receive IMC irradiation was investigated. Second, the impact of the addition of ipsilateral SCF irradiation on incidental IMNI dose was evaluated.

## Materials and Methods

### Patient Population

Between April 2012 and May 2017, patients who had undergone MRM or BCS and ALN dissection were included in this retrospective study. Patients who had undergone a sentinel node biopsy followed by an axillary dissection in the case of a positive node were also included. All these patients were newly diagnosed with histologically confirmed invasive breast carcinoma.

All patients were confirmed to have no clinical or pathological evidence of IMN involvement at the time of diagnosis, and IMCs were not included in the clinical target volume (CTV). The Institutional Research Ethics Board of Shandong Cancer Hospital and Institute approved this study (SDTHEC201703014), and all methods were performed in accordance with relevant guidelines and regulations. The requirement for written informed consent from patients was waived due to the retrospective nature of the investigation (retrospective single-institution cohort study).

### IMC Delineation

IMC CTV was defined by a radiation oncologist. IMC was delineated based on the Radiation Therapy Oncology Group (RTOG) breast cancer contouring atlas (online at: https://www.nrgoncology.org/Portals/0/Scientific%20Program/CIRO/BreastCancerAtlas_corr.pdf?ver=WoGzc4ixtKknUz1-6bVFCw%3d%3d), from ICS1–3 through the topography of the internal thoracic vessels. The planning target volume (PTV) of the IMC (PTV_IMC_) was designed to include an expansion of 5 mm around the IMC CTV. The same contouring atlas was followed to minimize the interobserver variability in the IMC and achieve the most precise and objective comparison.

### Treatment Planning

For patients undergoing MRM, the prescription dose to the PTV was 50 Gy in 25 fractions (2 Gy per fraction). For patients undergoing BCS, the prescription dose was 60.2 Gy in 28 fractions (2.15 Gy per fraction) to the PTV of the tumor bed and 50.4 Gy in 28 fractions (1.8 Gy per fraction) to the PTV of the breast. The enrolled patients were treated with one of the three irradiation techniques described below. All treatments were performed using 6-MV photon beams 5 days a week for 5–6 weeks.

#### Three-Dimensional Conformal Radiotherapy (3D-CRT)

The chest wall (breast) was treated with two opposite tangential fields, and the ipsilateral SCF was treated with a single anterior field. The criterion of the three-dimensional conformal radiotherapy (3D-CRT) plan was to ensure that at least 90% of the PTV received the prescription dose.

#### Field-in-Field Forward Intensity-Modulated Radiotherapy

The chest wall (breast) treatment plan involved the tangential field technique with static multileaf collimator segments and two parallel-opposed tangential fields. Two to five segmented fields were manipulated to maintain dose delivery to organs at risk, such as the ipsilateral lung and heart, within normally accepted tolerances and to reduce the volume of hot spots in the treatment field. Four to five fields were directed toward the SCF to guarantee dose uniformity. The criterion of the forward intensity-modulated radiotherapy (F-IMRT) plan was to ensure that at least 95% of the PTV received the prescription dose.

#### Inverse IMRT

The common isocenter was located at the center of the PTV. The tangential field technique was set to involve the entire PTV, and additional 0° and 40° MLC segments were constructed to involve the SCF. Additional subfields were set to reduce hot regions generated by the primary tangential fields and improve PTV dose uniformity to achieve dose homogeneity.

### Statistical Methods

Statistical analysis was performed with the SPSS statistical analysis software package. Based on the normality of the distributions, the Mann–Whitney *U* test was used to assess the statistical significance of the differences between the covariates. The Spearman rank correlation test was used to assess the relationship between IMNI dose differences and the covariates. All tests were two-sided. The results were regarded as statistically significant when *P* was <0.05.

## Results

Between 2012 and 2017, a total of 348 breast cancer patients were enrolled in this retrospective study. Among these, 138 patients received adjuvant postmastectomy radiotherapy, and the remaining 210 underwent radiotherapy after BCS. A total of 335 patients were diagnosed with invasive ductal carcinoma, 3 with invasive lobular carcinoma, 1 with invasive papillary carcinoma, and 9 with ductal carcinoma *in situ*. **Table 1** outlines the patient and treatment characteristics. None of the patients received radiotherapy to the ipsilateral IMC.

**Table 1:** Patient characteristics and treatment variables.

Characteristics	*n*	%
Age (years)		
Minimum	23	
Maximum	74	
Median	45	
Histology		
Invasive ductal carcinoma	335	96.26%
Invasive lobular carcinoma	3	0.86%
Invasive papillary carcinoma	1	0.29%
Ductal carcinoma *in situ*	9	2.59%
Tumor location		
Left-sided	154	44.25%
Right-sided	194	55.75%
Surgical		
MRM	138	39.66%
BCS	210	60.34%
Radiotherapy		
3D-CRT	118	33.90%
F-IMRT	119	34.20%
I-IMRT	111	31.90%
PTV		
Chest wall (breast)	217	62.36%
Chest wall + SCF	131	37.64%

The mean dose (Dmean) to the IMC was 29.69 Gy (range: 2.76–52.93 Gy) for all patients. According to the surgery employed, the Dmean to the IMC in patients undergoing MRM showed a greater coverage than in those undergoing BCS (32.85 vs. 27.10 Gy, *P* = 0.001). The incidental ICS1 dosimetry was also higher in the MRM group than in the BCS group. The differences in total IMC and ICS1–3 between the MRM and BCS surgical types while using different irradiation techniques are listed in **Table 2**.

**Table 2:** Comparison of the incidental IMC dose between the MRM and BCS groups.

	IMC	ICS1	ICS2	ICS3
All patients				
MRM	32.58 (2.76–50.93)	26.6 (4.83–48.18)	34.63 (4.06–51.71)	37.41 (3.46–54.7)
BCS	27.10 (4.09–52.93)	12.5 (1.69–54.29)	30.42 (3.65–63.13)	36.24 (4.79–57.91)
* Z*	−3.327	−6.922	−2.777	−1.103
* P*	0.001	0.000	0.005	0.270
3D-CRT				
MRM	33.8 (12.89–50.93)	27.78 (4.83–48.18)	36.42 (10.4–53.25)	38.43 (12.12–54.7)
BCS	26.84 (4.09–52.93)	11.4 (1.69–54.29)	30.82 (3.65–63.13)	34.5 (4.85–57.66)
* Z*	−2.328	−3.791	−1.978	−1.277
* P*	0.020	0.000	0.048	0.202
F-IMRT				
MRM	29.65 (2.76–46.64)	24.96 (6.85–50.27)	34.35 (4.06–51.7)	34.57 (4.79–57.91)
BCS	27.33 (4.12–51.40)	13.05 (1.77–50.68)	30.83 (3.72–61.18)	35.74 (4.79–57.91)
* Z*	−1.128	−3.537	−0.859	−0.005
* P*	0.259	0.000	0.391	0.996
I-IMRT				
MRM	32.95 (15.28–8.33)	26.49 (7.70–45.01)	34.65 (15.77–51.25)	39.21 (12.56–51.79)
BCS	26.5 (8.33–47.52)	12.68 (2.30–50.02)	30.04 (8.52–54.35)	38.13 (8.42–57.46)
* Z*	−2.407	−5.047	−1.833	−0.577
* P*	0.016	0.000	0.067	0.564

IMC, internal mammary chain; MRM, modified radical mastectomy; BCS, breast-conserving surgery.

**Figure 1:**
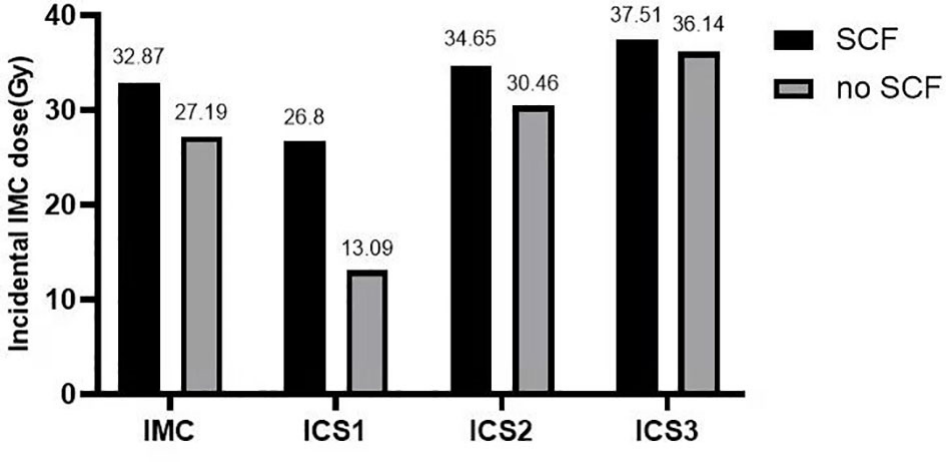
Histogram: mean incidental dose to the IMC and the first to third intercostal spaces of the IMC. The dose delivered to the IMC and the first two ICSs showed greater coverage in patients with SCF irradiation (*P* = 0.001, 0.000, 0.011). IMC, internal mammary chain; SCF, supraclavicular fossa; ICS1, first intercostal space; ICS2, second intercostal space; ICS3, third intercostal space.

Furthermore, 131 of the 348 patients (37.64%) received adjuvant postsurgical radiotherapy to the chest wall (breast) and ipsilateral SCF, and the remaining 217 (62.36%) received radiotherapy to the chest wall (breast) only. Except for patients who underwent F-IMRT, the average dose delivered to the IMC showed a greater coverage in the chest wall (breast) and ipsilateral SCF irradiation compared with only breast irradiation (**Table 3**). The dose delivered to the first two ICSs showed a greater coverage in patients with SCF irradiation than in those without SCF irradiation (**Figure 1**), whereas there was no significant difference for ICS3 (*P* = 0.296). Analysis of the patients who received 3D-CRT and inverse IMRT (I-IMRT) revealed that the Dmean of the IMC was also higher in patients with chest wall (breast) and ipsilateral SCF irradiation compared with only breast irradiation (*P* = 0.008, *P* = 0.016). However, there was no significant difference among patients who underwent F-IMRT (*P* = 0.407). For patients who underwent simultaneous integrated boost IMRT after BCS, the Dmean to the IMC in patients with inner-quadrant cancers showed a greater coverage of IMC than in patients with outer-quadrant cancers (**Table 4**). Similarly, the influence of the techniques was insignificant.

**Table 3:** Comparison of the mean dose in IMC with SCF versus without SCF.

	SCF		No SCF		P
	*N*	IMC (Gy)	*N*	IMC (Gy)	
All	131	32.87	217	27.19	0.001
3D-CRT	45	34.10	73	26.44	0.008
F-IMRT	46	29.20	73	27.46	0.407
I-IMRT	40	32.95	71	26.45	0.016

IMC, internal mammary chain; SCF, supraclavicular fossa.

**Table 4:** Impact of the location of the tumor bed on the incidental dose to the IMC.

	Location of the tumor (Gy)			*P*	
	Inner quadrants	Center quadrants	Outer quadrants		
3D-CRT	34.42	26.49	20.95	Inner vs. center	0.063
	(6.29–52.93)	(8.92–46.42)	(4.09–49.39)	Inner vs. outer	0.003
				Center vs. outer	0.315
F-IMRT	35.34	27.46	21.30	Inner vs. center	0.068
	(6.35–51.4)	(8.72–47.17)	(4.12–47.24)	Inner vs. outer	0.004
				Center vs. outer	0.366
I-IMRT	34.50	24.66	23.41	Inner vs. center	0.011
	(8.38–44.68)	(13.91–40.83)	(8.33–47.52)	Inner vs. outer	0.018
				Center vs. outer	0.920

IMC, internal mammary chain.

We evaluated the IMC coverage in patients treated after surgery, according to the type of surgery and whether the ipsilateral SCF was included in the irradiation field. In the univariate analysis, the incidental IMC dose was significantly higher in patients who underwent MRM and SCF irradiation. In the multivariate analysis, only the method of surgery was the correlative factor that affected incidental IMNI dose (**Table 5**).

**Table 5:** Univariate and multivariate regression analyses of incidental IMC dose difference.

Characteristic	Univariate analysis		Multivariate analysis	
	*r*	*P*-value	Coefficient (SE)	*P*-value
Surgery method	−0.175	0.001	−3.534	0.000
Ipsilateral SCF irradiation	−0.179	0.001		

SCF, supraclavicular fossa; IMC, internal mammary chain.

## Discussion

During radiotherapy for breast cancer, the rates of major coronary events increased linearly with the mean heart dose (MHD) (24–26), and for every 1-Gy increase in MHD, the Dmean of the left anterior descending artery increased by 3.4 Gy (27). The Breast Cancer Expert Panel of the German Society of Radiation Oncology (DEGRO) recommends an MHD <2.5 Gy for breast cancer radiation therapy (RT) treatment planning (28, 29). In patients with left-sided breast cancer who received IMC irradiation, the MHD was increased by nearly 3.5 Gy when compared with patients who did not receive IMC irradiation (8 vs. 5.6 Gy) (30). For left-sided breast cancer patients, the cumulative risk of cardiac deaths was 1.9% after 10 years, but it significantly increased to 6.4% after 20 years (29). The risk of breast cancer-specific mortality and a patient’s cardiac risk factors must be individually weighed against the risk of radiation-induced cardiotoxicity. DeSelm et al. (31) mapped the anatomic pattern of isolated nodal recurrences (NRs) in breast cancer patients treated with suitable surgery with or without RT, and 153 eligible patients were enrolled. Among the 79 NRs in the IMN chain, 63.3% (50/79), 18% (14/79), and 13% (10/79) were located in the first, second, and third ICSs, respectively. According to the guidelines of the RTOG or European Society for Radiation therapy and Oncology (ESTRO), there were 97.5% (77/79) IMN recurrences in the CTV. After curative system treatment, the overall recurrence rate in the IMNs was <1.5% even when the IMC was not excised or irradiated (9–12). Therefore, studies on the contribution of incidental radiation doses to the IMC are still ongoing. The purpose of these studies is to combine the physical and technical parameters, as well as genomic and radiomic characteristics, to improve the forecast performance of IMN metastasis and to prevent low-risk patients from receiving unnecessary radiotherapy that necessitates tailored system treatment (surgery, chemotherapy, endocrinotherapy, or targeted therapy).

Regardless of the radiotherapy technique, incidental ICS1 dose is higher in patients who have undergone MRM than in those who have undergone BCS (**Table 2**). The result of the correlation analysis showed that surgical treatment could affect the incidental IMC dose. Sapienza et al. found that the dose delivered to the IMC showed no significant difference between MRM and MRM plus immediate reconstruction (16). Hence, for patients treated with semi-opposed tangential 3D-CRT or IMRT, the incidental dose delivered to the IMC was not associated with chest wall thickness after MRM. The presternal fat thickness was inversely correlated with IMC inclusion in the tangent fields (32), and further analysis of dosimetric parameters proved that the higher volume of the PTV_IMC_ receiving a radiation dose of 40 Gy (V_40_) was correlated with a thin covering of presternal fat (33).

For patients treated with 3D-CRT after surgery, the Dmean to the IMC showed a greater coverage in patients who underwent MRM than in those who underwent BCS (20, 32, 34). Currently, IMRT has become the mainstream technology for treating breast cancer patients after surgery. A single−institute dosimetric study proved that the IMLN area receives higher incidental radiation dose for MRM than BCS in carcinoma breast patients treated with the F-IMRT technique (34). Patients with breast cancer who underwent postsurgery I-IMRT or F-IMRT were included in this study, and the calculated results have the same trend as the experimental results in patients who underwent 3D-CRT and I-IMRT. However, when patients underwent F-IMRT, the dose delivered to the IMC showed no significant difference between the MRM and BCS groups. To reduce OAR (e.g., ipsilateral lung, contralateral breast, heart) exposure, tomotherapy actively relocates isodoses from OAR areas toward areas usually with no restraint of the treatment plan optimization, such as the IMLN and ALN regions. Therefore, compared with 3D-CRT, the IMC incidental dose increased 106% during irradiation using the tomotherapy technique (13.5 vs. 27.8 Gy) (35). However, the tomotherapy technique has not become a routine radiotherapy option for breast cancer adjuvant radiotherapy.

With systematic and tailored therapy, an increasing number of breast cancer patients accept BCS directly or undergo BCS after neoadjuvant therapy (36, 37). Breast conservation versus mastectomy is associated with improved cosmetic outcomes and quality of life (38). Our previous study has found that a boost to the tumor bed in these patients did not increase the Dmean to the IMC, whether they underwent 3D-CRT, F-IMRT, or I-IMRT (21), which was consistent with the results of Sapienza et al. (the patient accepted CRT alone) (16). The location of the tumor bed is also a significant factor that influences IMC coverage. Regardless of the radiotherapy technique, a higher dose to the tumor bed increases the Dmean to the IMC in patients with inner-quadrant cancers.

According to the literature, SCF involvement is an insignificant predictor of IMC involvement dose (16, 23). In patients who received CRT, the incidental dose delivered to the IMC showed similar coverage in the SCF group compared with the non-SCF group (16). Therefore, it will be far more meaningful to study incidental IMC irradiation in a treatment plan that includes ipsilateral breast and supra/infraclavicular field. Our previous study found that when the influence of techniques was combined in patients with MRM, there was no significant increase in the Dmean to the IMC due to the addition of SCF (23). However, these two studies included a relatively small number of patients with SCF irradiation (nine and seven patients, respectively). The effect of regional lymphatic drainage area irradiation on the incidental IMC dose needs to be improved by increasing the sample size. A total of 131 patients with SCF irradiation were included in this study, and the dose delivered to the IMC showed a greater coverage of IMC in the SCF irradiation group compared with the non-SCF irradiation group (32.87 vs. 26.80 Gy). According to the correlation analysis, the irradiation field with or without SCF was not a parameter that affected the incidental Dmean of the IMC. The anatomic difference resulting from surgery type was the only parameter that affected IMC dose. According to our results, the addition of SCF irradiation increased the Dmean to the IMC in the 3D-CRT and I-IMRT groups, but not in the F-IMRT group (**Table 3**). Profound differences in the radiotherapy technique are most likely because for patients who underwent F-IMRT, SCF planning used half-beam irradiation (three to four fields), with the isocenter placed at the interface of the SCF field and chest wall/breast field. Furthermore, to reduce the apex pulmonis dose, the SCF field was irradiated with X-ray mixed with electron beams. Therefore, SCF plans were optimized to deliver at least 90% of the PTV receiving the prescription dose, which was below the planning acceptance criteria of 3D-CRT and I-IMRT.

The Early Breast Cancer Trialists’ Collaborative Group (EBCTCG) analyzed the long-term outcomes of neoadjuvant versus adjuvant chemotherapy in early breast cancer from 10 randomized trials and found that patients who received neoadjuvant chemotherapy (NACT) had a higher 15-year local recurrence after BCS than patients with the same dimensions who did not receive NACT (21.4% vs. 15.9%) (39). Thus, despite the increase in the feasibility of BCS for locally advanced breast cancer patients after NACT, to avoid underdosage in the IMC fields in patients who meet certain eligibility criteria (according to both preoperative clinical stage and postoperative pathological stage) and are indicated for elective IMNI, avoiding IMC irradiation using any of the above three techniques after BCS is not recommended.

## Conclusions

IMC received inadequate incidental radiation dose coverage with all the three techniques (3D-CRT, F-IMRT, and I-IMRT), both in patients undergoing MRM and BCS. The incidental dose delivered to the IMC was significantly lower in patients undergoing BCS than in those undergoing MRM, especially for the first ICS. We concluded that the most important factor affecting the incidental IMC dose was not SCF irradiation but the operative approach.

## Data Availability Statement

Publicly available datasets were analyzed in this study. The datasets used and/or analyzed during the current study are available from the corresponding author on reasonable request: lijianbin@msn.com.

## Ethics Statement

Approval was obtained from the Institutional Research Ethics Board of the Shandong Tumor Hospital Ethics Committee (SDTHEC201703014).

## Author Contributions

WW and JL participated in the study design, contributed to the data collection, and drafted the manuscript. TS, YZ, MX, and QS participated in the treatment planning. YM and YS made important contributions in collecting and analyzing the data and in revising the content. All authors read and approved the final manuscript.

## Funding

This study received funding from the Natural Science Foundation of Shandong Province (No. ZR2020QH260), National Natural Science Foundation of China (No. 8217102734), Taishan Scholars Program of Shandong Province (No. ts 20190982), and Breast Disease Science Foundation of Shandong Province Medical Association (No. YXH2020ZX062).

## Conflict of Interest

The authors declare that the research was conducted in the absence of any commercial or financial relationships that could be construed as a potential conflict of interest.

## Publisher’s Note

All claims expressed in this article are solely those of the authors and do not necessarily represent those of their affiliated organizations, or those of the publisher, the editors and the reviewers. Any product that may be evaluated in this article, or claim that may be made by its manufacturer, is not guaranteed or endorsed by the publisher.
